# Qualities of Children’s Fear in Therapeutic Action Groups Addressing Post-separation Parental Stalking

**DOI:** 10.1177/13591045221136638

**Published:** 2022-11-05

**Authors:** Anna Nikupeteri, Pia Marttala, Merja Laitinen

**Affiliations:** 1 Faculty of Social Sciences, 7003 University of Lapland, Rovaniemi, Finland; 2 Private provider of psychotherapy services through the Social Insurance Institution of Finland, Finland

**Keywords:** fear, stalking, post-separation period, children, therapeutic group intervention, trauma psychotherapy

## Abstract

This paper examines the way in which parental stalking — as a form of domestic abuse — raises fear in children and affects their sense of safety. The study draws on three therapeutic action groups involving 13 children who have experienced stalking by their fathers/stepfathers after the parents’ separation. The research question is as follows: How does children’s sense of fear manifest in therapeutic action groups? The qualitative analysis revealed three qualities of fear among the children: (1) internalised, (2) constant and (3) episodic. *Internalised fear* appeared as a child’s mental state that materialised as an overwhelming sentiment in the group sessions and elsewhere. *Constant fear* activated at times, and the senses of fear and security alternated both in the sessions and elsewhere. *Episodic fear* related to the children’s memories of violent events and father’s stalking behaviour. The children were able to sense security in the group and in daily life owing to a temporal distance to their father’s stalking. Our findings underscore the importance of professionals’ awareness of the qualities of children’s fear and the significance of assessing their fear and sense of safety in a child-centered manner in therapeutic practices.

## Introduction

Fear is one of the main consequences of violence in children’s close relationships (e.g. Arai et al., 2021; Noble-Carr et al., 2020; Pernebo & Almqvist, 2017; Øverlien, 2013; 2014). Despite the established knowledge of fear among the victims, nuanced knowledge of children’s fear is scarce. The purpose of this paper is to address children’s sense of fear by focusing on therapeutic action groups offered for children exposed to their father’s/stepfather’s stalking of the mother after separation.

In this article, parental stalking is approached as a form of violence against women and children. Stalking is typically understood as pursuit-oriented, violent or nonviolent action consisting of repeated, intrusive, coercive and controlling behaviours — such as following, harassing and threatening — that cause fear and distress in victims (e.g. Logan & Walker, 2017; Spitzberg & Cupach, 2014). Thus, it is not necessarily the behaviours themselves that are violent, but the context in which they are used (Logan & Walker, 2017). The main target of stalking is usually the child’s mother, but the father also often uses children as a means to get in contact with the ex-partner, or as direct targets of violent acts and threats (Løkkegaard et al., 2019; Nikupeteri & Laitinen, 2015). Nowadays the use of technological devices and online media increases the complexity of stalking by making the perpetrator “omnipresent” (Nikupeteri et al., 2021). According to Elklit et al. (2019), parental stalking causes complex, multiple-event and continuous trauma in children. In their study of 57 children, 56% met the diagnostic criteria for post-traumatic stress disorder.

Fear plays a crucial role in stalking, and fear as the victim’s emotional response to the perpetrator’s behaviour is involved in many definitions and laws concerning stalking (Dietz & Martin, 2007; Reyns & Englebrecht, 2013). Some laws state that stalking victimisation requires a person to be frightened by stalking, while others state that it requires a reasonable person to experience fear because of the activity. A person may not be defined as a victim if one has not feared for one’s own safety or the safety of the immediate family, if one has not been afraid of being harmed or getting killed, or if one has not suffered from emotional distress and apprehension (e.g. Logan & Walker, 2017; Spitzberg & Cupach, 2014). The nature of stalking as repeated action and the dynamic nature of fear, which may change over time, create an additional challenge in assessing a victim’s fear (Reyns & Englebrecht, 2013). Children’s sense of fear is accentuated by children’s dependence on adults for daily care (Øverlien, 2013). The fear factor required in stalking victimisation creates a unique ground for examining children’s fears.

This study draws on three piloting therapeutic action groups for children exposed to stalking perpetrated by the father/stepfather. The children were approached as agents who are capable of reflecting on their experiences of violence if adults respect and facilitate their right to participation (Callaghan, Fellin, Mavrou, et al., 2017; Eriksson & Näsman, 2012; Swanston et al., 2014; Øverlien, 2014). The purpose of the therapeutic group intervention was to offer the children a possibility to share their experiences of stalking with peers and to give them possibilities and tools to feel safe under parental stalking. The children’s sense of fear was approached as an emotional response triggered by cognitive processes, as anxiety or stress and as a perception that one is at risk of victimisation or feels unsafe (Reyns & Englebrecht, 2013).

The aim of the paper is to contribute to academic discussions and therapeutic practice on how fear plays out in children’s experiences of post-separation parental stalking and how it can be addressed in therapeutic group interventions for children. The research question is: How does children’s sense of fear manifest in therapeutic action groups? To our knowledge, this is the first study elaborately addressing the fears of children who are exposed to domestic abuse, particularly post-separation parental stalking.

## Methodology

This study belongs to a larger Finnish project Children's Knowing Agency in Private, Multiprofessional and Societal Settings - The Case of Parental Stalking investigating children’s experiences and agency in parental stalking. As a part of the project, professionals in the national Stalking Support Center piloted three therapeutic action groups for children. The professionals, who were experienced in individual and group work with children exposed to domestic abuse, contacted families meeting the following criteria: (1) The clienthood of the parent and child/children had lasted a long time, (2) the child was or had been a client in his/her own right and (3) the child was otherwise a suitable participant considering the family situation or the child’s age and stage of traumatisation. To gather information about the family situation, the professionals met the children and their mothers and fathers with joint custody separately before the group sessions started. The second author of this paper acted as a professional in two groups and all three authors analysed the material.

Altogether 13 children (8 girls and 5 boys) aged 2–12 from seven families participated in the three groups (Table 1). The children were gathered into groups of three to five based on their sibling relations and experiences. Some of them were stalked directly, whereas others were used as a means of stalking. Many of the children were still in contact with their father at least through supervised meetings.

**Table 1.** Groups and their participants.

Group number	Gender	Age
Group 1 (G1)	Boy	12
	Boy	12
	Girl	10
	Girl	8
	Girl	7
Group 2 (G2)	Boy	8
	Boy	6
	Girl	9
	Girl	2
	Girl	9
Group 3 (G3)	Boy	9
	Girl	12
	Girl	6

The groups were based on the first phase of trauma-related psychotherapy, stabilisation, the aim of which was to help the children to engender a feeling of safety, practice skills, create relationships with safe adults, and alleviate the symptoms of trauma (Steele et al., 2017) prior to engaging in the trauma resolution work. Each group had from eight to ten sessions and they met once a week, comprising 28 sessions altogether. The duration of each session was 90 minutes and they were videotaped. The professionals reflected on some of the meetings and these discussions were also recorded. The group sessions were not transcribed, as it was not possible to adequately document the action and simultaneous talk in them.

### Ethical Considerations

The research was approved [no. 278/00.05/2016] by the Research Ethics Committee of the University of Lapland . Throughout the research process we committed to the ethical guidelines of the World Medical Association Declaration of Helsinki (2018). Two local associations of the collaborating Stalking Support Center approved the research permit. Permission from both parents was requested when the parents had joint custody, and the children and their guardians gave their informed, written consent. In the case of the two-year-old participant, the parents gave their written consent on behalf of the child. The children had the possibility to withdraw from the group during any phase of the process.

### Analysis

The analysis was content-oriented (Mason, 2007). The group sessions enabled us to watch and hear not only the children’s reports on situations in which they were “frightened”, but also their expressions of fear during action and interaction (Reyns & Englebrecht, 2013). We were interested in how fear manifested in the groups, how the children expressed it and how they dealt with it. First, we thematised the sources of the children’s fears, their fear-related events, their behaviours towards fears, their ways of expressing fear and their coping methods. Second, we evaluated the children’s fear-related behaviour and the intensity of their accounts and expressions. Finally, we identified three qualities of fear among the children: (1) internalised, (2) constant and (3) episodic. Next, we will describe the principles and structure of the group sessions and elaborate on the qualities of fear. We illustrate the findings through anonymised examples from the children and professionals.

## Principles and Structure of the Therapeutic Action Groups

The professionals carefully planned the principles and structure of the groups and designed the activities for each group based on their evaluation of what would be most beneficial for the children. The key themes of the groups were fear, (in)security, parents’ separation and violence, but there were also positive themes such as resources, wishes and coping methods. Since the work dealt with the intensity of children’s fear and (in)security, it was necessary to place two or three professionals in each group.

The professionals utilised both established and creative methods in eliciting the children’s experiences. Each group meeting started with refreshments and ended with relaxation. The activities between them varied, including drawing, painting, discussion, making a collage, and listening to music, stories and drama. The children also suggested some activities themselves. In one group, the activities were related to the elements earth, fire, air and water. The professionals tailored exercises to the children after assessing their reactions and tolerance, and they were ready to stop the activities if needed. The children were allowed to participate in the activities as much as they wanted (Fellin et al., 2019). The two-year-old child had partly her own activities. Each group had a special closing session including a celebration of some type: eating pizza, inviting the mothers to the group, or watching a fire performance outside.

The starting point in the groups was that the children were able to feel confident and secure both physically and in their disclosures (Callaghan, Fellin, Mavrou, et al., 2017). This was achieved by getting to know one another, defining shared group rules and agreeing on the group structure. A space of trust, respect and safety was also built by physical means, for example by creating a safe space in the room encircled by yarn (“safety yarn”). The children were not allowed to talk about other children’s issues outside the group. The children knew that the professionals and the fathers had agreed that the fathers will not interrogate the children about the issues discussed in the group. Overall, the groups adhered the ethics of care, protection and participation (Eriksson & Näsman, 2012).

## Qualities of Children’s Fear in Post-Separation Parental Stalking

**Figure 1.**
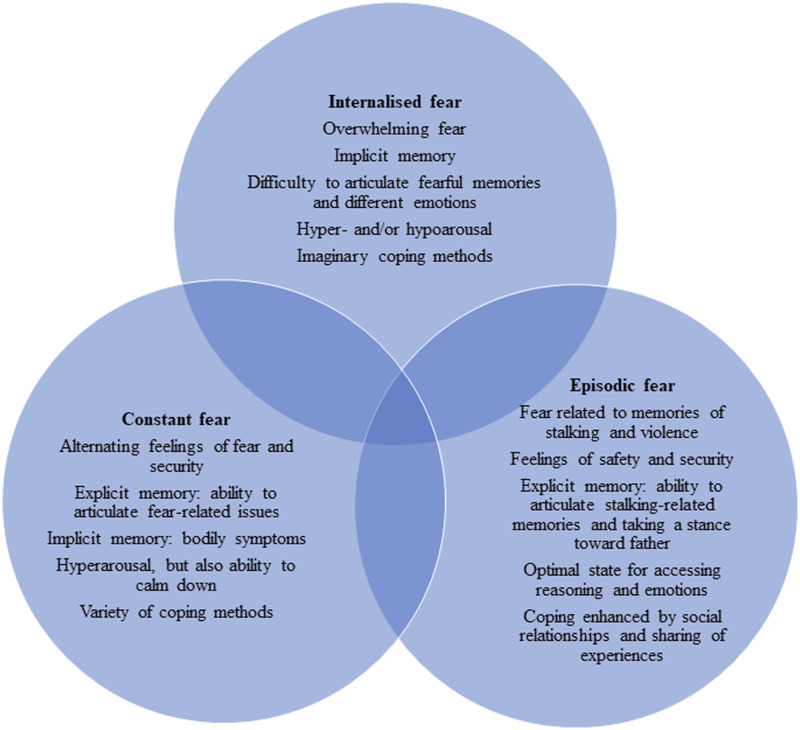
Qualities of fear in the children.

We identified three qualities of fear in the children through the therapeutic action group sessions (see Figure 1).

### Internalised Fear

Internalised fear was a fearful state where fear was overwhelming (also Swanston et al., 2014). The trauma reminders were related to everything in the children’s daily lives, and the threat of violence and the father’s unpredictable behaviour made them observe their environment and stay constantly alert (Nikupeteri & Laitinen, 2015; Øverlien, 2013). Based on the group sessions, the fear manifested as unconscious, nonverbal and bodily tension expressed implicitly by the children.

In the sessions, the children’s fear materialised as hyper- and inactivity. Hyperarousal appeared as physical restlessness and hypoarousal as regression (Ogden & Fisher, 2015). The fear was triggered by any external issue, such as exchanging news of the week in the beginning of the session by choosing a bear card that represented one’s own feelings. In hypoarousal, the children could not recognise or describe their emotions even in tasks involving symbolic elements, such as drawing while listening to music chosen to arouse basic emotions. The children benefitted from mindfulness exercises (Ogden & Fisher, 2015), where the children activated their senses for example by having refreshments in the beginning of the session:

It can be calming to concentrate on how the food tastes and feels… Take your time to think how it feels in your mouth, how it tastes*.* (Professional)

The children with internalised fear had difficulty distinguishing between a real and an imagined threat of violence. Some of the children’s fears were universal in nature, such as the fear for being at home alone or in the dark, constituting an integral part of child development and representing an innate need for protection by adults (Taimalu et al., 2007). However, considering their age, the children’s fear was overpowering. The other fears narrated by the children were imagined, involving for instance monsters and zombies (Drawing 1. The Bogeyman). The children located their fears in different places (also Fellin et al., 2019). One child drew a picture of the Devil sitting in the sauna (Drawing 2. Devil). A nine-year-old boy (G3) told that in his ears he hears the voice of a door opening, and that he spends time after school hiding behind the freezer and watching the door, expecting his father to step in at any moment. In one session (G3), siblings discussed fears:


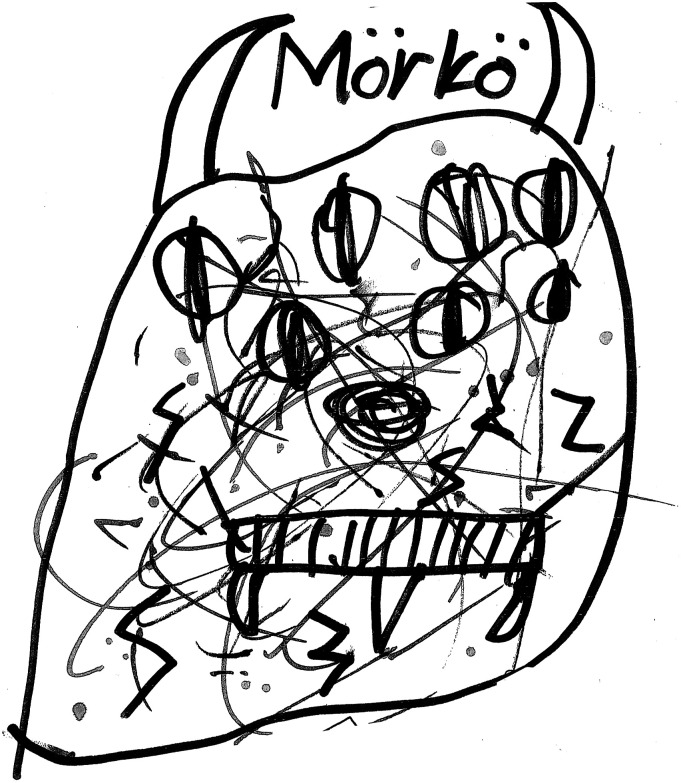


**Drawing 1.** The Bogeyman.


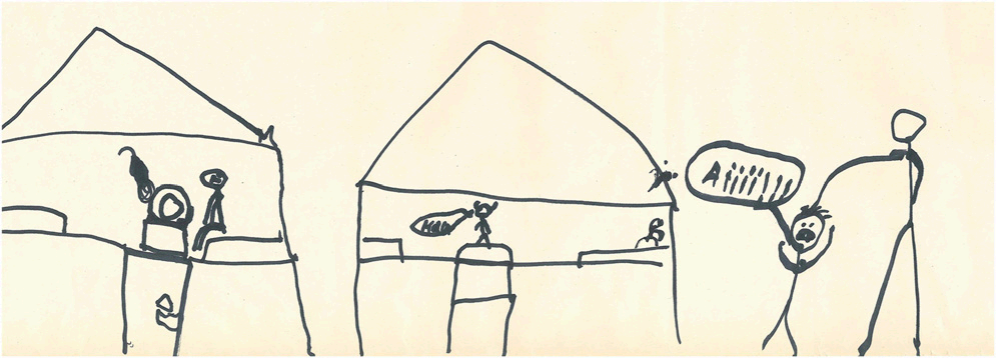


**Drawing 2.** Devil.

[My brother] is afraid every day when he comes home. (Girl, 12 years)

Actually, a safe haven is needed… At least I need one*.* (Boy, 9 years)

The children with internalised fear lacked violence-related words and had difficulty articulating violence-related issues (also Arai et al., 2021). For example, a ten-year-old girl (G1) could not recall any bad events related to family live. When discussing the supervised meetings with her father, she said that “*there is a supervisor*”, but she could not describe her feelings related to these meetings. This occurred regardless of the creation of a safe space by encircling a part of the room with “safety yarn” to give the children a possibility to think freely and to be oneself in a space marked off for that purpose (also Callaghan, Fellin, Mavrou, et al., 2017). The children’s coping methods were often imaginary, such as using magic or weapons such as tripwire or bombs, and they escaped into a fantasy world. One child described his defense methods (G3):

I imagine that I put a weapon in my backpack…a hammer. (Boy, 9 years)

The children’s fear was characterised by worry (also Noble-Carr et al., 2020). In two groups, the children wrote their worries on a piece of paper and put it in a stuffed animal with a large mouth and belly, the “Worry Gobbler”, which ate the worries and freed the children from them during the session. The children also discussed their worries:

Daddy frightens mum… that he appears at the door. (Boy, 9 years, G3)

Mom is afraid that we [children] get killed. (Girl, 9 years, G2)

The children with internalised fear had difficulty naming safe places or people. Thus, engendering safety by material means was important to them (also Fellin et al., 2019). In one group (G2), the children felted safety animals. In another group (G3), they constructed their own safety box by making a collage in a large cardboard box. They were instructed to draw inside the box things that increased their safety. The children drew for example a rainbow and a stuffed animal, and even wrote “fuck off”. On the box they were instructed to describe issues that protected them. These were, for example, the text “Be kind and go away”, a drawing of a karate kick, and tripwire placed outside the box.

The children with internalised fear lived with repeatedly activating fear in the group and outside of it. The activities for these children were aimed at ameliorating the pain and dysfunction caused by recurring trauma and at preventing retraumatisation (Schamess, 1993). To achieve this, the professionals were to respect the children’s constrained capacity for agency and their need to manage disclosures in order to stay safe (Callaghan, Fellin, Mavrou, et al., 2017). In establishing safety, it was important that the professionals supported the children in regulating their sense of fear and insecurity (Fellin et al., 2019; Steele et al., 2017). The children benefitted from action that enabled them to calm down and enhanced their sense of security in the group.

### Constant Fear

Constant fear was related to actual stalking events and threats, but it also allowed the children to feel safe. The children experienced fear and insecurity implicitly and as bodily symptoms (also Callaghan, Fellin, Alexander, et al., 2017). The sense of fear was in the core, although the children discussed other emotions too, such as anger, sorrow and frustration. They were able to recall violent events, to narrate their violence- and stalking-related experiences and to share them with other children.

The children with constant fear at times experienced hyperarousal in the sessions. Their fear activated by mental images or outer triggers. Even good memories related to father and his good-natured contacts induced fear, as the children related these to father’s bad behaviour. A nine-year-old boy (G3) found it a “*very bad thing*” when his father said he would move closer to the children and when the father once visited his school. A six-year-old girl said that it felt bad when her father found out which kindergarten she goes to. However, the children were able to manage their fear and calm down through stabilising bodily exercises, discussions with other children and professionals, and engagement in imaginary activities not related to their personal experiences.

Many of the children with constant fear were interested in hearing about and discussing the stories of other children. Other children thereby played a crucial role in trying to understand one’s own experiences (Fellin et al., 2019; Schamess, 1993). For example, a seven-year-old girl (G1) returned several times to issues that stirred up her fear, such as her parent being intoxicated and having unsupervised meetings with the father. Fear as a shared experience made the children curious about one another’s experiences (Fellin et al., 2019). In one session (G1), a girl and a boy discussed fears:

Were you afraid? (Girl, 7 years)

I’m usually not afraid of anybody. (Boy, 12 years)

Surely all people are afraid of something? (Girl, 7 years)

Yeah, a little bit… Don’t have that kind of fear, except maybe one. (Boy, 12 years)

The professionals followed the children’s dialogues carefully. At times they had to interrupt the discussions to minimise confrontation and to normalise potentially painful emotions, such as an induced sense of insecurity (Schamess, 1993). For example, in the discussion above the professionals explained that the boy need not be afraid every day. In this case, the professionals assumed the role of a “protective symbolic parent” (Schamess, 1993) and asserted their autonomy as adults (Bunston et al., 2016). Sometimes other children’s stories may have recurred in the children’s minds, as narrated by one of the children (G1):

Do those stories still bother some of us, like, if they have some memories about the issue, does it still bother them? (Girl, 7 years)

In stressful situations, the professionals instructed the children to sense their body (also Van der Kolk, 2014). The children were offered blankets and a possibility to roll a stone or stress ball in hand to calm them down. In one session (G1), a professional prompted the children to think how they feel stress and fear in their body:

If something sticks to your mind, it doesn’t necessarily bother you as a thought. Instead you may feel it in your body for instance as tension in shoulders or an upset stomach or you may feel sick or you might breathe differently, right? How does your body react when something bothers you or sticks to your mind? (Professional)

I feel it in my head and chest. (Boy, 12 years)

Discussing coping methods and comparing them with others helped the children to process their coping strategies (also Fellin et al., 2019). The children used a variety of coping methods, including imaginary, physical and subtle ones. Some children, for example, discussed scary events with their mothers, made a safe haven in bed at home or stayed overnight at a friend’s house after a threatening situation. A seven-year-old girl (G1) described a changeover from mother to father; she purposefully prolonged her staying with the mother at a gas station in order to avoid meeting the father.

Despite father’s stalking, the children with constant fear were able to feel secure both in the sessions and elsewhere, as they perceived that the fear was related to actual events and threats. The children’s sense of security fluctuated and they were able to use coping methods. Participation in the groups significantly supported their coping and enabled them to engender safety and to regulate their fear through stabilising exercises and discussions with peers.

### Episodic Fear

In terms of episodic fear, there was a mental, emotional and temporal distance between the children and stalking-related events, although the fathers still stalked their mothers. The children did not meet their father or there had been a long time since they met him last. They could feel secure in the sessions and their daily life and were able to narrate and share their experiences by combining verbal and non-verbal expressions.

In the sessions, the children were in an optimal state as to being able to use both reasoning and emotions. They were able to use their cognitive abilities, handle traumatic memories and regulate their sense of fear and insecurity. Discussing past experiences activated their fear, but the explicit memories enabled them to control themselves, as they understood that the violent experiences belonged to the past (Ogden & Fisher, 2015). The children were also able to calm down and relax unprompted if they noticed that they were tense (also Callaghan, Fellin, Alexander, et al., 2017). For example, one child (G1) noted while rolling a stone in her hand:

Im annoyed, that’s why I take the stone in my hand. (Girl, 7 years)

The children narrated their experiences using violence-related words. In one session (G1), the theme was to discuss how children define violence. The children wrote and drew their thoughts on sticky notes and fastened them to a clothesline with clothes pegs. The children referred to violence for instance as follows: “coercion”, “when somebody tries to threaten the other”, “hitting”, “you are not allowed to attack a person” and “calling somebody different”. They also drew an exclamation mark and a teardrop. As the task activated negative memories in the children, the professional instructed them to do bodily exercises and to feed the “Worry Gobbler” with the sticky notes in order to restore their sense of safety:

Let these issues go, the sad issues have been laundered now, let’s use some friendly soap and leave the sad issues behind [washing her body] and the Worry Gobbler can go ahead and make them vanish for good. (Professional)

The children with episodic fear were able to evaluate their family relations and discerned different aspects in their family situations. Some of them took a stance and were critical towards their father’s stalking behaviour. One child (G1) perceived her father’s behavior as wrong and violent and expressed a wish concerning their family life:

I have such a mean dad. Why doesn’t he listen, even though he’s been told so many times. (…) It’s also a bit annoying that so many people have kind of an ordinary family… so it’d be nice just to have an ordinary family [referring to her neighbour]. (Girl, 7 years)

Discussing their family relations enabled the children to grasp the complexity and also positive sides of their family life and social networks. This even applied to the perpetrating father (also Fellin et al., 2019). One of the tasks was to create, by means of bricolage, a tree of social relations where apples represented the children’s good relations and lemons stood for difficult ones. In making the tree, some children had ambivalent feelings about their father, as reflected on by a boy when trying to decide whether an apple or a lemon would represent the father best:

There has been some apple to it, too, sometimes [thinking about father’s good sides], but I still won’t put an apple there… That lemon [father’s bad sides] is actually my father. The one I have to meet… and then I should discuss [with father]. I actually go to meet him but... well I do go to meet him, but I just tell him exactly what I think because otherwise he won’t listen*.* (Boy, 12 years)

Developing resources and assuring children’s own coping were central elements for children with episodic fear (Fellin et al., 2019; Ogden & Fisher, 2015). The professionals supported the children’s positive self-identity for instance by giving them a certificate depicting their personal strengths. In terms of coping, the children valued their social relations and the possibility to share experiences with other children. The children proved to be actors who are not afraid to act (Øverlien, 2014), as stated by two girls (G1):

I was tired of listening to mum and stepfather arguing, so I often could not take it, so if somebody gets angry or starts to yell, I try to protect the others, so I took my brother and little sister to our room or to my room, and we stayed there until it was quiet. (Girl, 8 years)

Well that's a good thing*.* (Girl, 7 years)

The children benefitted from the psychoeducational approach in the group (also Schamess, 1993). The professionals listened to the children, and when they observed that the children pondered certain issues, they provided related information for example on violence, stalking or intoxicants. In one of the sessions, the children and the professionals discussed children’s rights by utilising cards focusing on the issue (Photo 1. Children’s rights cards) with an aim to add the children’s knowledge of their rights as citizens and to strengthen their agency. The professionals told the children about the symptoms and roots of trauma, and about adaptive action after a threatening situation or when faced with negative memories (Schamess, 1993). They also enhanced the children’s understanding of their fear responses:


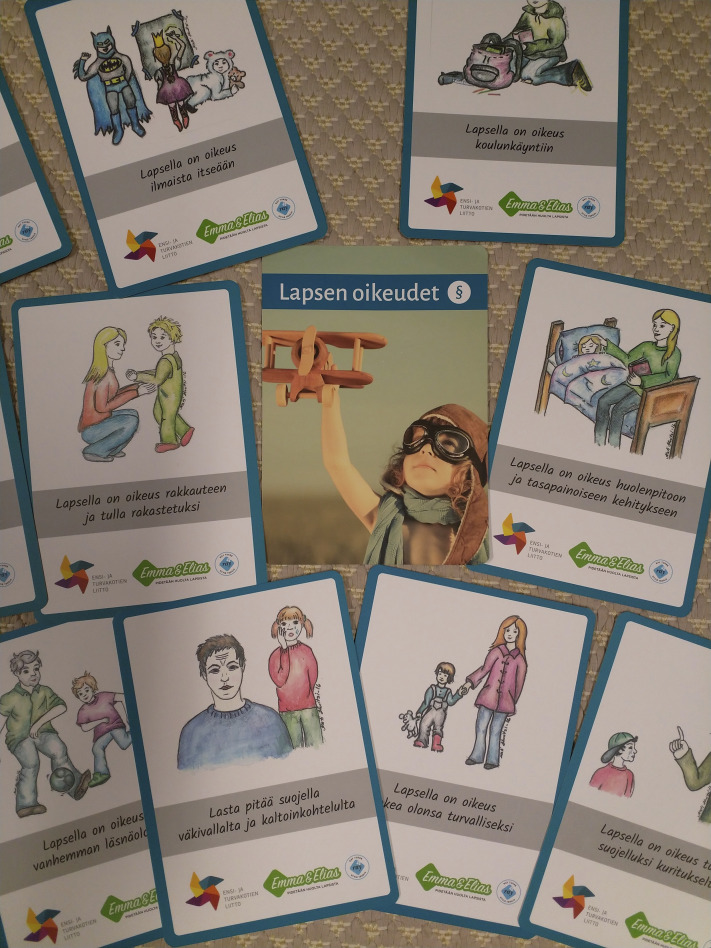


**Photo 1.** Children's rights cards.

And especially, if one really has had those kinds of experiences, with a real reason to be afraid, then one is even more prone to be afraid or becomes frightened also in situations where there is no real threat. (Professional)

On the whole, the children with episodic fear benefitted most from the therapeutic group intervention in that they were able to identify and develop their personal strengths and resources with peers. The professionals could also follow the structure of the group process from addressing the challenges to focusing on the children’s resources. The children had the ability to construct a resilient sense of self in relation to their father’s stalking behaviour (also Fellin et al., 2019).

## Identifying and Working on the Different Qualities of Children’s Fear

Drawing on the children’s therapeutic action groups, our analysis shows that children’s sense of fear entails three qualities. *Internalised* fear manifested as an overwhelming emotion and was constantly present in the group and elsewhere. This quality of fear is caused by a child’s exposure to father’s severe and prolonged stalking (see also Arai et al., 2021). The children could experience a feeling of safety in the group, but it did not extend to their daily lives. Regarding *constant* fear, the sense of fear and security alternated both in the sessions and in daily life. The fear was situation-related and based on the actual stalking, but the children were able to use defensive methods. In *episodic* fear, there was a mental, emotional and temporal distance between the children and stalking, and the children were able to feel secure in the group and outside of it. Overall, the qualities of fear were not static and the intensity of fear varied in the children’s lives.

Our findings echo the earlier ones on domestic abuse as to how children manage their emotional experiences in embodied, relational and contextually relevant ways (Callaghan, Fellin, Alexander, et al., 2017; Øverlien, 2013). The data indicate that children disclose their experiences of parental stalking in group sessions to a varying extent depending on their developmental stage (Arai et al., 2021), the severity and intensity of stalking in their lives, and the nature of their relationship with their father. It is therefore important to offer children a range of ways to express their experiences verbally and non-verbally (Fellin et al., 2019). Moreover, the pilot group arrangement emphasises the importance of risk assessment and safety planning when referring children to therapy.

The findings highlight a contextualised and relational understanding of children’s sense of fear (also Callaghan, Fellin, Alexander, et al., 2017). The intensity of fear may vary even between children in the same family, which strengthens the earlier findings that stalking victims’ fear varies according to situational and victim characteristics (Dietz & Martin, 2007; Reyns & Englebrecht, 2013). It is important that professionals assess children’s quality of fear in order to determine the appropriate form of help. For example, in terms of children with constant fear, limiting or suspending contacts with the stalking father may relieve their uneasiness. When aiming to strengthen children’s sense of safety and to engender safety in their physical and social circumstances (Steele et al., 2017), it is important to make child-led safety plans. Identifying the quality of children’s fear requires long-term therapeutic work with them.

There are several limitations on offering therapeutic action groups for the children. In our pilot groups, the age range was rather wide and the children were at different stages in terms of sensing fear in their daily lives. Gathering together children in different stages of development and with varying intensity of fear may pose a risk if those experiencing strong fear raise the fear level of others whose family situation may be more secure (also Fellin et al., 2019). However, our work indicates that it is important to offer children a possibility for group therapy focusing on stalking: While children with internalised fear may get an understanding of protection, children with episodic fear get a possibility to develop their resources further. In order to help children more comprehensively in the future, it would be important to offer a parallel group for mothers who are stalked by their ex-partners (Bunston et al., 2016).

The study increases our knowledge of how children may sense fear when they are exposed to stalking and, more broadly, to domestic abuse (Dietz & Martin, 2007; Reyns & Englebrecht, 2013). Based on our findings, professionals are to identify and consider the different qualities of fear among children in order to develop appropriate interventions: On the one hand, a child may need to be protected from a stalking father and supported in creating physical safety. On the other hand, it may be possible to address the negative consequences of the father’s action through therapeutic intervention. Professionals’ awareness of the qualities of children’s fear and their expertise to assess it are key components in responding to children’s need for help.
